# Supplementary material for: DRAXIN regulates interhemispheric fissure remodelling to influence the extent of corpus callosum formation
Source: eLife. 2021 May 4;10:e61618. doi: 10.7554/eLife.61618 (PMC8137145; doi:10.7554/eLife.61618)
Supplement: Supplementary file 1. [file elife-61618-supp1.docx]

Supplementary File 1: Statistics

| **Figure** | **Data compared** | **Number of samples** | **Statistical test** | **p value** |
| --- | --- | --- | --- | --- |
| 1C | HC length cCCD vs pCCD | n = 27 cCCD, n = 15 pCCD | Ordinary one-way ANOVA with post Tukey’s multiple comparisons test | 0.0154 |
| 1C | HC length cCCD vs full CC | n = 27 cCCD, n = 66 full CC | Ordinary one-way ANOVA with post Tukey’s multiple comparisons test | < 0.0001 |
| 1C | HC length pCCD vs full CC | n = 15 pCCD, n = 66 full CC | Ordinary one-way ANOVA with post Tukey’s multiple comparisons test | 0.0767 |
| 3D | Ratio dorso-ventral IHF length pCCD humans | n = 9 control, n = 10 pCCD | Unpaired t test | 0.0044 |
| 3E | Ratio dorso-ventral CC width pCCD humans | n = 9 control, n = 10 pCCD | Mann-Whitney test | 0.0322 |
| 3G | Ratio anterior-posterior IHF length anterior segment pCCD humans | n = 9 control, n = 8 pCCD | Unpaired t test | 0.0023 |
| 3H | Ratio anterior-posterior IHF length posterior segment pCCD humans | n = 9 control, n = 8 pCCD | Unpaired t test | <0.0001 |
| 3I | Ratio anterior-posterior CC length anterior segment pCCD humans | n = 9 control, n = 10 pCCD | Unpaired t test | 0.0029 |
| 3J | Ratio anterior-posterior CC length posterior segment pCCD humans | n = 9 control, n = 10 pCCD | Mann-Whitney test | < 0.0001 |
| 5A | CC length chr4:rs6397070 SNP | n = 10 heterozygous, n = 21 homozygous | Kruskal-Wallis ANOVA with Dunn’s multiple comparison | 0.0006 |
| 5B | HC length chr4:rs6397070 SNP | n = 10 heterozygous, n = 21 homozygous | Kruskal-Wallis ANOVA with Dunn’s multiple comparison test | > 0.9999 |
| 5C | AC area chr4:rs6397070 SNP | n = 10 heterozygous, n = 21 homozygous | Kruskal-Wallis ANOVA with Dunn’s multiple comparison test | > 0.9999 |
| 5E | CC length chr9:rs29890894 SNP | n = 15 heterozygous, n = 16 homozygous | Kruskal-Wallis ANOVA with Dunn’s multiple comparison | > 0.9999 |
| 5F | HC length chr9:rs29890894 SNP | n = 15 heterozygous, n = 16 homozygous | Kruskal-Wallis ANOVA with Dunn’s multiple comparison test | > 0.9999 |
| 5G | AC area chr9:rs29890894 SNP | n = 15 heterozygous, n = 16 homozygous | Kruskal-Wallis ANOVA with Dunn’s multiple comparison test | > 0.9999 |
| 5I | CC length chr15:rs31781085 SNP | n = 16 heterozygous, n = 15 homozygous | Kruskal-Wallis ANOVA with Dunn’s multiple comparison | > 0.9999 |
| 5J | HC length chr15:rs31781085 SNP | n = 16 heterozygous, n = 15 homozygous | Kruskal-Wallis ANOVA with Dunn’s multiple comparison test | > 0.9999 |
| 5K | AC area chr15:rs31781085 SNP | n = 16 heterozygous, n = 15 homozygous | Kruskal-Wallis ANOVA with Dunn’s multiple comparison test | > 0.9999 |
| 5M | CC length *Draxin* mutation | n = 39 heterozygous, n = 29 homozygous | Kruskal-Wallis ANOVA with Dunn’s multiple comparison test | 0.0002 |
| 5N | HC length *Draxin* mutation | n = 39 heterozygous, n = 29 homozygous | Kruskal-Wallis ANOVA with Dunn’s multiple comparison test | > 0.9999 |
| 5O | AC area *Draxin* mutation | n = 39 heterozygous, n = 29 homozygous | Kruskal-Wallis ANOVA with Dunn’s multiple comparison test | > 0.9999 |
| 6B | Ratio IHF length ventral forebrain C57 versus BTBR | n = 6 C57, n = 6 BTBR | Mann-Whitney test | 0.0152 |
| 6B | Ratio IHF length mid-horizontal forebrain C57 versus BTBR | n = 6 C57, n = 6 BTBR | Mann-Whitney test | 0.0022 |
| 6B | Ratio IHF length dorsal forebrain C57 versus BTBR | n = 6 C57, n = 6 BTBR | Mann-Whitney test | 0.0022 |
| 6E | Ratio IHF width C57 versus BTBR | n = 7 C57, n = 6 BTBR | Mann-Whitney test | 0.0012 |
| 6G | NESTIN fluorescence intensity ROSTRAL 0 – 50 μm lateral to the IHF, C57 versus BTBR | n = 7 C57, n = 6 BTBR | Mann-Whitney test | 0.4452 |
| 6G | NESTIN fluorescence intensity ROSTRAL 50 – 100 μm lateral to the IHF, C57 versus BTBR | n = 7 C57, n = 6 BTBR | Mann-Whitney test | 0.0221 |
| 6H | NESTIN fluorescence intensity CAUDAL 0 – 100 μm lateral to the IHF, C57 versus BTBR | n = 7 C57, n = 6 BTBR | Mann-Whitney test | 0.4452 |
| 6K | E14 SOX9-positive MZG, C57 versus BTBR | n = 9 C57, n = 8 BTBR | Unpaired t test | 0.0003 |
| 6K | E15 SOX9-positive MZG, C57 versus BTBR | n = 6 C57, n = 6 BTBR | Mann-Whitney test | 0.0022 |
| 6L | E15 SOX9-positive MZG 0-50 μm bin, C57 versus BTBR | n = 6 C57, n = 6 BTBR | 2-way ANOVA with Sidak’s multiple comparison test | 0.0002 |
| 6L | E15 SOX9-positive MZG 50-100 μm bin, C57 versus BTBR | n = 6 C57, n = 6 BTBR | 2-way ANOVA with Sidak’s multiple comparison test | <0.0001 |
| 6L | E15 SOX9-positive MZG 100-150 μm bin, C57 versus BTBR | n = 6 C57, n = 6 BTBR | 2-way ANOVA with Sidak’s multiple comparison test | 0.0003 |
| 6L | E15 SOX9-positive MZG 150-200 μm bin, C57 versus BTBR | n = 6 C57, n = 6 BTBR | 2-way ANOVA with Sidak’s multiple comparison test | 0.0072 |
| 6L | E15 SOX9-positive MZG 200-250 μm bin, C57 versus BTBR | n = 6 C57, n = 6 BTBR | 2-way ANOVA with Sidak’s multiple comparison test | 0.8128 |
| 6L | E15 SOX9-positive MZG 250-300 μm bin, C57 versus BTBR | n = 6 C57, n = 6 BTBR | 2-way ANOVA with Sidak’s multiple comparison test | >0.9999 |
| 8E | Total midline length E13 C57 versus BTBR | n = 7 C57, n = 8 BTBR | Mann-Whitney test | 0.1520 |
| 8E | Total midline length E14 C57 versus BTBR | n = 9 C57, n = 8 BTBR | Unpaired t test | 0.9152 |
| 8E | Total midline length E15 C57 versus BTBR | n = 4 C57, n = 8 BTBR | Unpaired t test | 0.5507 |
| 8F | Percent EdU-positive/DAPI-positive MZG E13 | n = 6 C57, n = 8 BTBR | Mann-Whitney test | 0.9497 |
| 8F | Percent EdU-positive/DAPI-positive MZG E14 | n = 9 C57, n = 7 BTBR | Mann-Whitney test | 0.0115 |
| 8F | Percent EdU-positive/DAPI-positive MZG E15 | n = 4 C57, n = 7 BTBR | Mann-Whitney test | 0.0121 |
| 8G | Percent EdU-positive/KI67-positive MZG E13 | n = 6 C57, n = 8 BTBR | Mann-Whitney test | 0.5728 |
| 8G | Percent EdU-positive/KI67-positive MZG E14 | n = 9 C57, n = 7 BTBR | Mann-Whitney test | 0.1142 |
| 8G | Percent EdU-positive/KI67-positive MZG E15 | n = 4 C57, n = 7 BTBR | Mann-Whitney test | 0.0061 |
| 8H | Percent EdU-positive/KI67-negative MZG E13 | n = 6 C57, n = 8 BTBR | Mann-Whitney test | 0.5728 |
| 8H | Percent EdU-positive/KI67-negative MZG E14 | n = 9 C57, n = 7 BTBR | Mann-Whitney test | 0.1142 |
| 8H | Percent EdU-positive/KI67-negative MZG E15 | n = 4 C57, n = 7 BTBR | Mann-Whitney test | 0.0061 |
| 8I | EdU-positive cells IHF base E13 | n = 7 C57, n = 8 BTBR | Mann-Whitney test | 0.0003 |
| 8I | EdU-positive cells IHF base E14 | n = 9 C57, n = 7 BTBR | Mann-Whitney test | 0.0086 |
| 8I | EdU-positive cells IHF base E15 | n = 4 C57, n = 7 BTBR | Mann-Whitney test | 0.1636 |
| 8J | Density EdU-positive/KI67-positive cells IHF base E13 | n = 7 C57, n = 8 BTBR | Mann-Whitney test | 0.0007 |
| 8J | Density EdU-positive/KI67-positive cells IHF base E14 | n = 9 C57, n = 7 BTBR | Mann-Whitney test | 0.7577 |
| 8J | Density EdU-positive/KI67-positive cells IHF base E15 | n = 4 C57, n = 7 BTBR | Mann-Whitney test | 0.4121 |
| 8K | Density EdU-positive/KI67-negative cells IHF base E13 | n = 7 C57, n = 8 BTBR | Mann-Whitney test | 0.3450 |
| 8K | Density EdU-positive/KI67-negative cells IHF base E14 | n = 9 C57, n = 7 BTBR | Mann-Whitney test | 0.0934 |
| 8K | Density EdU-positive/KI67-negative cells IHF base E15 | n = 4 C57, n = 7 BTBR | Mann-Whitney test | 0.0727 |
| 3-S1D | Ratio anterior-posterior fused septum length pCCD humans | n = 9 control, n = 6 pCCD | Mann-Whitney test | 0.0044 |
| 8-S1 | E13 volume base IHF C57 versus BTBR | C57 n = 7, BTBR n = 8 | Mann-Whitney test | 0.014 |
| 8-S1 | E14 volume base IHF C57 versus BTBR | C57 n = 10, BTBR n = 6 | Mann-Whitney test | 0.016 |
| 8-S1 | E15 volume base IHF C57 versus BTBR | C57 n = 4, BTBR n = 7 | Mann-Whitney test | 0.0121 |

AC = anterior commissure, CC = corpus callosum, cCCD = complete corpus callosum dysgenesis, Chr = chromosome, HC = hippocampal commissure, IHF = interhemispheric fissure, MZG = midline zipper glia, pCCD = partial corpus callosum dysgenesis, SNP = single nucleotide polymorphism.
